# Why you should not skip tailored exercise interventions when using incretin mimetics for weight loss

**DOI:** 10.3389/fendo.2024.1449653

**Published:** 2024-07-23

**Authors:** Katharina Gross, Christian Brinkmann

**Affiliations:** ^1^ Department of Preventive and Rehabilitative Sport Medicine, Institute of Cardiovascular Research and Sport Medicine, German Sport University Cologne, Cologne, Germany; ^2^ Department of Fitness & Health, IST University of Applied Sciences, Düsseldorf, Germany

**Keywords:** incretin, GLP-1, obesity, weight loss, exercise, training

## 1 Introduction

Modern incretin mimetics are a hot topic in the treatment of adults with obesity or overweight (1). Weekly subcutaneous injections of glucagon-like peptide-1 (GLP-1) receptor agonists (RAs) or dual GLP-1/glucose-dependent insulinotropic polypeptide (GIP) RAs have been shown to elicit weight reductions of up to 15–20% in adults with obesity or overweight (2, 3) – levels of weight loss previously reported only after bariatric surgery. The effects of GLP-1 RAs are manifold: they increase postprandial insulin secretion from pancreatic β-cells, suppress glucagon secretion from α-cells, slow gastric emptying, and reduce appetite. The suppression of appetite under GLP-1 RA treatment is presumably induced by actions on peripheral vagal nerve endings in the gut mucosa that project into the central nervous system, engaging satiation signals. Whether and how incretin mimetics can access central brain targets relevant for appetite suppression is a subject of current debate (4). While the precise mechanisms behind the hypophagic effects of GLP-1 RAs are still not fully understood, GLP-1 RAs like liraglutide or semaglutide, as well as tirzepatide, a GLP-1/GIP RA, have been authorized for weight loss as an adjunct therapy to lifestyle interventions in adults with obesity (body mass index (BMI) of ≥ 30 mg/m²) or adults with overweight (BMI ≥ 27 mg/m² but ≤ 30 mg/m²) who have at least one weight-related health problem. However, in practice, diet and exercise may risk being overshadowed by what has been described as a “miracle drug” in public media. Here, we present some reasons why regular exercise is crucial during and after incretin-based pharmacotherapy for people who want to lose weight.

## 2 Why exercise during and after incretin-based pharmacotherapy for weight loss?

### 2.1 Exercise can help prevent the loss of muscle mass

A recently published systematic review on the effects of semaglutide pharmacotherapy on body weight and composition reported that significant weight reductions were observed primarily due to a loss of fat mass (5). However, weight loss was also accompanied by a decrease in lean mass, with notable reductions of up to 40% of total weight reduction (significant effects were particularly evident in larger trials). The largest study included in this review, the Semaglutide Treatment Effect in People with Obesity (STEP-1) randomized clinical trial (RCT), demonstrated that semaglutide treatment not only elicited a significant loss of fat mass but also of lean body mass compared to placebo (-8.36 kg vs. -1.37 kg and -5.26 kg vs. -1.83 kg, respectively) (2).

Maintenance of functional muscle mass is pivotal for an individual’s health, as loss of muscle mass is associated with physical frailty, metabolic disturbances, and cardiovascular diseases (6). Skeletal muscle-derived secretory proteins (‘myokines’) can mediate cross-talks with other organs thereby inducing important positive health benefits. Therefore, the goal of maintaining muscle mass should be a priority. Notably, in the STEP-1 trial, monthly counseling sessions encouraging patients to increase their physical activity to 150 min per week with activities such as walking were not sufficient to prevent the loss of lean mass. This highlights that general counseling, when referring to physical activity guidelines, may not be very effective for this target group and that exercise interventions need to be more specific and supervised. For instance, Sardeli et al. showed in a meta-analysis that supervised resistance training at a minimum of 65% of one repetition maximum (1RM) for 12 to 24 weeks can reduce more than 90% of lean body mass loss during a calorie-restricted diet (7). These results demonstrate that targeted exercise interventions alongside weight loss therapies can successfully prevent skeletal muscle atrophic effects. This approach maximizes the muscle-to-fat ratio and enhances health benefits.

Very recently, it has been discussed to use muscle hypertrophy-stimulating drugs to counteract obesity and reductions in lean mass during weight loss (8). Injections with bimagrumab (compared to placebo) alongside diet and exercise counseling elicited significant skeletal muscle hypertrophy and reductions in fat mass over a 48-week period in a phase 2 clinical trial involving adults with obesity or overweight and concomitant type 2 diabetes mellitus (9). Moreover, glucose homeostasis improved with reductions in glycated hemoglobin (HbA1c) levels (9). Diarrhea and muscle spasms were the most frequently reported adverse events by patients in the bimagrumab group.

Bimagrumab inhibits the activin type-2 receptor, thereby preventing the binding of its ligands and blocking their potent anti-hypertrophic effects (10). Yet, a key question here is whether health benefits can be attributed to the sheer quantity of (inactive) skeletal muscle, or whether it is primarily skeletal muscle *contraction* that facilitates the physiological mechanisms behind the broad health benefits. Wackerhage et al. speculate that myokines might be the “prime candidates” to explain the mechanisms by which increased muscle mass can cause fat loss and improvements in glycemic control (8). The circulatory levels of several myokines are affected by the duration and/or intensity of physical activity (11, 12). Thus, here too, the active use of muscles would be an important factor contributing to better health.

### 2.2 Exercise can help reduce weight regain after terminating incretin-based pharmacotherapy

Nearly all patients experience weight regain after discontinuation of a GLP-1 mimetic weight loss program. Despite the large weight losses observed under semaglutide therapy in the STEP-1 trial, two-thirds of the mean weight lost over the 68 weeks were regained in the following year after the treatment was stopped (13). This raises the question of whether patients will therefore be dependent on the medication for the rest of their lives to maintain weight, with unanswered questions about long-term safety and efficacy, or whether there are other effective strategies to maintain weight loss.

Some studies indicate that sport and exercise are suitable for maintaining body weight or at least mitigating weight regain. In a study by Lundgren et al., 166 participants were randomized to a 52-week maintenance treatment with either liraglutide alone, liraglutide with supervised exercise, placebo alone or placebo with supervised exercise following an 8-week weight loss period (with a calorie-restricted diet). The combination therapy of liraglutide and supervised exercise led to approximately twice the decrease in body fat percentage compared to either liraglutide alone or placebo with exercise (14). It is noteworthy that the placebo with exercise intervention resulted in similar reductions in body fat as the treatment with liraglutide alone, each compared with placebo without exercise. Considering the common gastrointestinal side effects of GLP-1 RAs, and in light of concerns regarding overmedication in the elderly, exercise may be a better alternative than liraglutide treatment for weight loss maintenance.

More important results became apparent after a further follow-up period. After the 52-week interventions, all treatments stopped, and analyses continued 1 year later (week 104) (15). It is remarkable that in the groups that did exercise (accumulated data), the loss of fat mass from the diet phase (weeks -8 to 0) was maintained better (weeks 0–104) than in the groups that did not exercise. One year after terminating all treatments, participants in the exercise and the combined group still demonstrated the highest self-reported levels of physical activity with 240 min per week and 225 min per week, respectively, whilst the liraglutide group reported only 30 min per week. It appears that the supervised training had lasting effects on the patients’ physical activity behavior after the interventions were stopped.

A pivotal aspect of this trial is *how* the maintenance of an active lifestyle was achieved. The exercise intervention in the study by Lundgren et al. was structured and supervised (14). Wilding et al. only recommended their patients to move regularly and to follow the World Health Organization’s (WHO) guidelines (minimum of 150 min moderate-to-vigorous activity per week) alongside semaglutide treatment (2). In Lundgren et al.’s study, each participant was assigned to a qualified instructor with a bachelor’s or master’s degree in exercise physiology who planned and monitored the exercise program throughout the trial (14). Participants were encouraged to visit supervised group sessions with 45 min of vigorous-intensity twice per week and to perform moderate-to-vigorous-intensity exercise individually two more times per week. This exercise program resulted in adherence to the recommended exercise volume by the WHO of 119 ± 70% and 113 ± 71% for the exercise and the combination group, respectively, and in the aforementioned high levels of physical activity one year after terminating all treatments (14, 15). Unfortunately, Wilding et al. did not report the actual physical activity levels of their participants (2).

Current guidelines for obesity management state that clinicians should “encourage” and “advise” adults to increase physical activity (16). Yet, the aforementioned trials show that advice alone is not enough. Supervised, structured exercise programs are needed to maintain weight loss and sustainably reduce fat mass. Lifestyle interventions are the first-line treatment for weight management, and pharmacotherapy is only indicated after there has not been sufficient weight loss in a lifestyle intervention. Nonetheless, there is a great degree of heterogeneity in exercise programs that all fall under the umbrella term of lifestyle interventions. While the combination of pharmacotherapy and exercise remains the most successful therapy, supervised exercise programs have been shown to elicit similar results to pharmacotherapy alone (14) and come with fewer adverse health effects and better long-term outcomes (15). Thus, there is a need to explore what constitutes effective exercise interventions and how they can be implemented into clinical practice.

But how can regular exercise support weight loss maintenance?

The regulation of body weight by activation of exercise-induced signaling pathways is a highly complex process (17). Regular exercise can have beneficial effects on energy expenditure, fat oxidation, and regulation of food intake (18). From an energetic point of view, muscle mass and its utilization play a major role. On the one hand, exercise-induced changes in body composition (at the same absolute body weight) can raise resting metabolic rate (RMR). However, this effect should not be overestimated. Muscle mass has a RMR of just around 13 kcal/kg/day, while the RMR of fat mass is around 4.5 kcal/kg/day (18). On the other hand, there can be a notable increase in energy expenditure through additional physical activity/exercise – as extreme examples of elite cyclists in the grand tours demonstrate by expending roughly 8000 kcal per day (19).

However, it should be noted that appropriate nutritional advice may sometimes be useful, as exercise may lead to (over-)compensatory eating in some individuals (20). Additionally, care should be taken not to restrict other (non-exercise) activities in daily living during an exercise intervention to ensure a favorable energy balance (21).

### 2.3 Exercise can help reduce self-perception of poor health

Taking medication may reinforce the belief of feeling unwell, which has important implications. It has been reported that a negative rating of one’s own health is closely related to a higher prevalence of chronic diseases, mortality, and hospital visits (22). In a previous study, a negative subjective perception of one’s health status was associated with depressive symptoms (23). This perception may also lead to reduced engagement in health measures, thereby triggering a vicious cycle. In the study by Lundgren et al. (14), treatment with liraglutide or placebo significantly reduced the general health perception. On the flip side, adding exercise to the pharmacological intervention did not significantly change the patients’ general health perception; both exercise groups maintained their initial improvement in well-being after the first weight-loss maintenance phase. Exercise can, therefore, counteract the self-perception of poor health that can accompany the regular use of medication.

### 2.4 Exercise can help counteract increased resting heart rate from GLP-1 RA treatment

GLP-1 RA treatment has been shown to slightly increase resting heart rate (1). However, in the study by Lundgren et al. (14), significant increases in resting heart rate were not observed when exercise was added to incretin mimetic-based therapy. Being able to counteract this negative effect from GLP-1 RA treatment has important clinical implications, as an increased resting heart rate is a predictor for adverse outcomes, including mortality and development of diseases (24, 25).

### 2.5 Exercise can help improve health beyond weight loss

“Health is a state of complete physical, mental and social well-being and not merely the absence of disease or infirmity.” (26) Regular physical activity irrefutably contributes to the primary and secondary prevention of a broad range of chronic conditions, ranging from diabetes mellitus to cardiovascular disease (CVD), cancer, bone and joint disease, and depression (27, 28). At the same time, physical *in*activity is an independent risk factor for the development of obesity-related complications and comorbidities (29).

Physical activity not only aids weight loss but also simultaneously improves health across a spectrum that is broader than any medication has ever achieved. One of the most important effects is that it improves cardiorespiratory fitness, which can protect against the development of diseases. A high level of cardiorespiratory fitness is associated with reduced all-cause mortality and premature death from CVD (30). Incretin therapy does not improve cardiorespiratory fitness, but exercise interventions alone or in combination do (14).

## 3 Conclusive remarks

**Figure 1:**
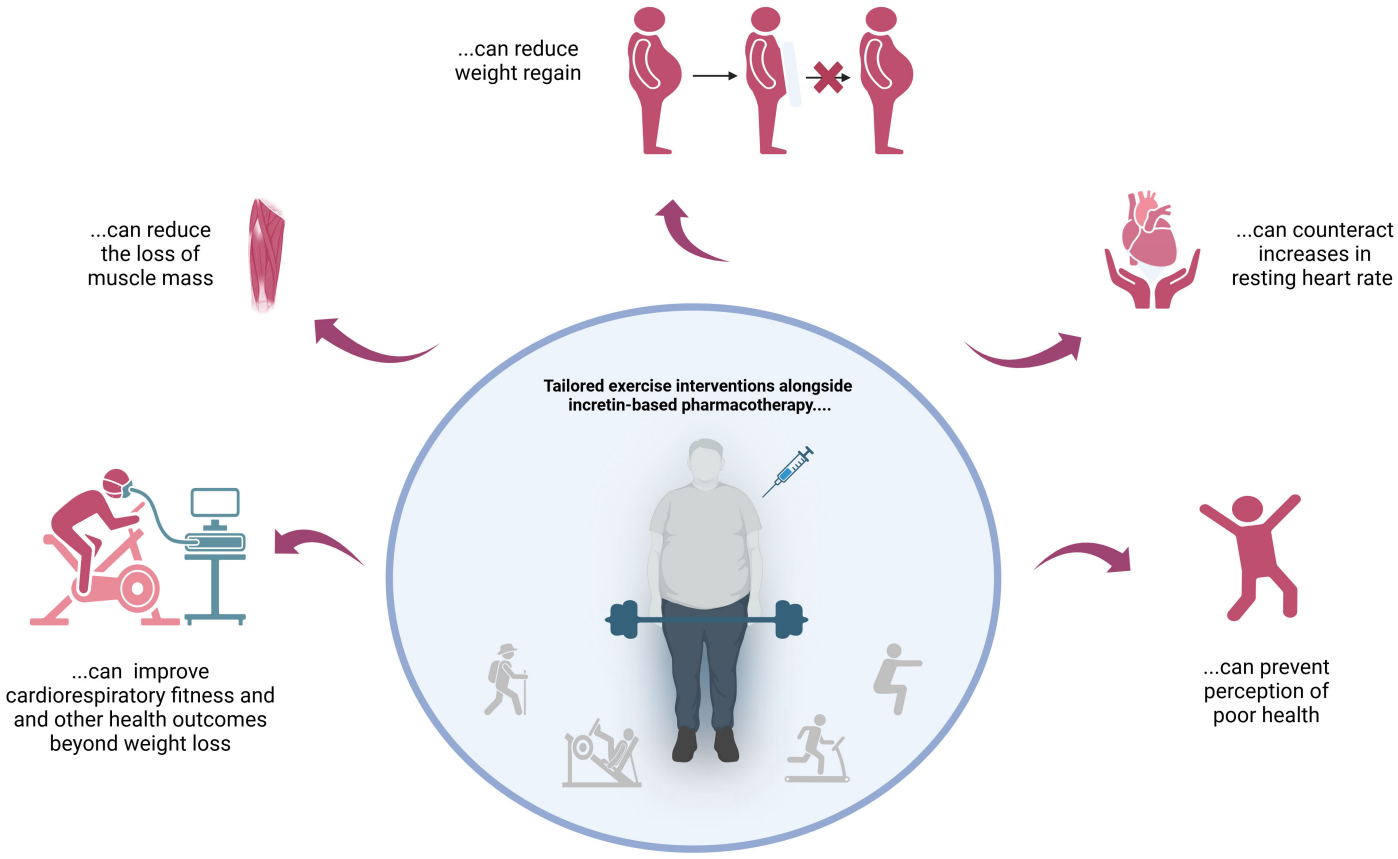
Reasons not to skip tailored exercise interventions when using incretin mimetics for weight loss.

The findings described above clearly suggest that incretin-based pharmacotherapy should always be combined with targeted exercise interventions – preferably right from the start! This approach counteracts undesirable side effects and improves outcomes in both the short and long term (**Figure 1**).

Advice on exercise alone is not very effective in changing patients’ activity behavior. Instead, the use of planned, supervised exercise programs is much more appropriate and has been shown to elicit similar reductions in body fat and weight as pharmacotherapy. In this context, the question of the optimal type, amount, and intensity of exercise arises. Further studies are needed to evaluate the effectiveness of supervised exercise training and weight loss programs that combine structured exercise with incretin mimetic therapy to further increase the evidence for the benefits of such interventions compared to pharmacotherapy alone (alongside lifestyle recommendations).

It is also important to change society’s and patients’ perceptions of the use of the so-called “miracle drug”. Relying solely on the drug is overly simplistic! Incretin mimetics are intended for people who cannot manage without medication and who are at risk of developing health problems. A holistic, multimodal approach to obesity therapy is needed, ensuring that patients receive comprehensive care at all levels.

## Author contributions

KG: Writing – review & editing, Writing – original draft, Visualization. CB: Writing – review & editing, Writing – original draft, Supervision.
